# Isolation and Purification of Antibacterial Compound from Kombucha of SCOBY

**DOI:** 10.4014/jmb.2504.04012

**Published:** 2025-08-12

**Authors:** Younhee Nam, Junghoon Lee, Soo Rin Kim, Jong Nam Kim

**Affiliations:** 1 Department of Food Science and Nutrition, Dongseo University, Busan 47011, Republic of Korea; 2 Division of Chemical Engineering, Dongseo University, Busan 47011, Republic of Korea; 3 School of Food Science and Biotechnology, Kyungpook National University, Daegu 41566, Republic of Korea; 4 Department of Advanced Bioconversion, Kyungpook National University, Daegu, 41566, Republic of Korea

**Keywords:** Antibacterial, kombucha, SCOBY, instrumental analysis, HMF

## Abstract

The misuse of antibiotics has contributed to the widespread emergence of antimicrobial resistance (AMR), emphasizing the need for alternative antimicrobial agents. Kombucha, a fermented beverage containing a symbiotic culture of bacteria and yeast (SCOBY), has gained attention for its antibacterial activity and potential health benefits. This study investigated the antibacterial properties of kombucha and SCOBY, isolating and characterizing the active compounds responsible for these effects. Both kombucha broth and dried SCOBY effectively inhibited *Escherichia coli*, *Listeria monocytogenes*, *Staphylococcus aureus*, and *Salmonella Typhimurium*, with dried SCOBY demonstrating stronger activity. Instrumental analyses identified 5-hydroxymethylfurfural (HMF) as the primary antibacterial compound in the SCOBY extracts. HMF significantly inhibited *L. monocytogenes* and *S. aureus*, with its antibacterial inhibition surpassing that of chloramphenicol in these two bacterial species. Previous studies have shown that, in addition to its antibacterial effects, HMF has potential applications in the production of polymers and pharmaceuticals, and as a fuel additive, suggesting its potential in the chemical and biofuel industries. This study highlights the antibacterial activity of HMF and underscores the need for further research to evaluate its safety and applicability in various fields.

## Introduction

Antibiotics are widely used to treat infections. However, recently, antimicrobial resistance (AMR) has emerged due to the excessive use of antibiotics [1]. Since the discovery of penicillin by Alexander Fleming in 1928, antibiotics have revolutionized infection treatment, especially after the 1940s, following advancements in penicillin purification by Chain and Florey. However, by the early 2000s, antibiotic-resistant strains had become increasingly prevalent. Since the development of new antibiotics is challenging due to technical and financial limitations, alternative infection treatments such as vaccines, antibodies, probiotics, bacteriophages, and antifungal yeasts are under investigation to address resistance arising from indiscriminate antibiotic use [2, 3].

A fermented beverage made by combining green or black tea with sugar and a symbiotic culture of bacteria and yeast (SCOBY), kombucha has recently attracted attention for its reported potential health benefits, which include reductions in cholesterol and blood pressure, and hepatoprotective, immunomodulatory, and gastrointestinal regulatory effects. However, further validation is required to confirm these outcomes [4, 5]. Additionally, some studies suggest that kombucha exhibits anticancer properties and significant antimicrobial activity, which may contribute to its growing use as a functional beverage [5, 6]. The reported antimicrobial activity of kombucha can be attributed to its organic acids and complex microbial consortia, which are increasingly being investigated for clinical or therapeutic use [6, 7]. Kombucha has been shown to exhibit antimicrobial activity against various pathogenic bacteria and fungi, such as *Staphylococcus epidermidis*, *Escherichia coli*, *Staphylococcus aureus*, and *Candida albicans* [8, 9]. This activity is maintained even after heating and filtration, suggesting that kombucha’s antimicrobial effects are inherent to the kombucha broth and not solely attributable to acetic acid bacteria (AAB) or yeast [6, 10]

SCOBY, which drives kombucha fermentation, is composed primarily of AAB and yeast and forms a new cellulose layer at the liquid–air interface during the fermentation process. This creates a carbon-rich, moist environment that supports the AAB growth necessary for cellulose production; however, fermentation also requires strong antimicrobial properties to counter potential microbial contamination [3, 11] . SCOBY is composed of bacterial nanocellulose and has a porous structure [12], which acts as a matrix that can retain various bioactive metabolites generated during fermentation, such as organic acids, polyphenols, and antimicrobial compounds. While SCOBY is not considered directly beneficial to human health, it functions as a reservoir for fermentation-derived metabolites and a scaffold for essential microbial communities. After kombucha production, SCOBY is typically regarded as food waste; however, due to its bioactive compound content and structural properties, it holds potential for repurposing in applications such as skincare and cosmetics [13, 14]

While the antibacterial properties of kombucha are well documented, the specific antibacterial attributes of SCOBY and the compounds responsible for these effects remain largely unexplored. Consequently, this study investigates the antibacterial properties of kombucha, aiming to isolate and characterize its active compounds through extraction and instrumental analysis.

## Materials and Methods

### Preparation of Kombucha

Green tea (*Camellia sinensis*; 4.8 g) was brewed in 800 ml of water at 95°C for 5 min. After removing the tea leaves, 100 g of sucrose (Junsei Chemical Co., Ltd., Japan) was added, followed by sterilization. Once cooled to room temperature, approximately 150 g of SCOBY and 200 ml of fermented kombucha broth were added. The final volume was adjusted to 1 L. The culture was then incubated at room temperature for 21 days, with subculturing performed at 21 days.

### Microbial Test Strains

Antimicrobial tests were conducted using four pathogenic bacterial strains, namely, *E. coli* ATCC 35150, *Listeria monocytogenes* EGD-e, *S. aureus* Newman, and *Salmonella typhimurium* SL1344. Pathogenic bacterial strains were cultured after inoculation into optimal media. *E. coli* and *L. monocytogenes* were inoculated into tryptic soy agar (BD, USA), and *S. aureus* and *S. typhimurium* were inoculated into Luria-Bertani agar (BD). Subcultures were used three times after activation by culturing at a culture temperature of 37°C for 16 h [15].

### Evaluation of Antibacterial Activity

The antibacterial activity assay followed methods from previous studies, with slight modifications based on disc-diffusion method [16]. Sterilized media were inoculated with bacteria at a concentration of 8 × 10^7^ CFU/ml and dispensed into petri dishes. Samples were then applied onto 8 mm paper discs (Advantec Co., Ltd., Japan) and dried at 40°C. The tested samples included a pH 2.3 adjusted solution (acetic acid; Junsei), fermented kombucha broth, dried SCOBY (ø 8 mm), methanolic extract of SCOBY, 35 μl/disc SCOBY extract fractions (SF), 30 μg/disc chloramphenicol (Merck, Germany), and 20 mg/disc 5-hydroxymethylfurfural (HMF; Merck). Each loaded disc was then placed onto the bacterial inoculated medium and incubated at 37°C for 16 h. Antibacterial activity was quantified by measuring the diameter of the clear inhibition zone (cm) around each disc.

### Antibacterial Compound Extraction

The SCOBY was washed twice with distilled water and subsequently dried at 60°C for 5 h, at 75°C for 5 h, and at 90°C for 48 h. The dried SCOBY was ground to the smallest possible particle size, and 54.75 g of the ground material was extracted with 1 L of methanol (Junsei, Japan). After a 24-h extraction period, the SCOBY residue was removed using a vacuum pump. The methanol was then entirely evaporated at 65°C using a rotary evaporator (V-700 vacuum pump, R-215 rotavapor system, B-491 heating bath; Switzerland) to obtain a concentrated extract paste [17]. The methanolic extract was subsequently subjected to silica gel column chromatography. A silica gel column (2 × 28 cm) was packed and sequentially eluted with acetonitrile (Honeywell, USA), followed by an acetonitrile-water gradient with increasing polarity. Sequential elution was performed by adding 50 ml each of 95%, 70%, and 50% acetonitrile–water solutions. Fractions were collected at 1 ml intervals, and the inhibition zone diameter was measured. Only fractions with a confirmed inhibition zone were retained. To further purify these fractions, they were filtered through a 0.45 μm filter, and the acetonitrile–water solutions were then removed using a rotary evaporator.

### Instrumental Analysis of Antibacterial Agent

**Fourier-transform infrared spectroscopy (FT-IR).** After mixing the sample with methanol at a 1:1 ratio, 100 μl of the mixture was drop-cast onto a KBr pellet (Edmund, USA) following spin-coating. Fourier-transform infrared spectroscopy (FT-IR; IRAffinity-1; Shimadzu Co., Japan) was performed in transmission mode. Spectra were recorded over 20 scans within the range of 4,000–400 cm^-1^. Before sample measurement, the KBr pellet was calibrated to account for atmospheric CO_2_ and humidity. Baseline correction was conducted using the Origin software (ver. 2019b), and peaks were analyzed to identify functional groups.

**Gas chromatography–mass spectrometry (GC/MS).** Gas chromatography–mass spectrometry (GC/MS; 450-GC, 320-MS; Bruker, USA) was performed using a DB-5MS column(30 m × 0.25 mm ID × 0.25 μm film thickness; Agilent Technologies, USA) with helium as the carrier gas. The GC oven was initially held at 50°C for 3 min before being ramped to 300°C at 5°C/min and then maintained at 300°C for 3 min. The inlet split ratio was set to 10:1, and mass spectra were acquired through electron ionization, scanning a mass range of 35–450 m/z.

**X-ray photoelectron spectroscopy (XPS).** X-ray photoelectron spectroscopy (XPS; NEXSA; Thermo Fisher Scientific, USA) analysis was conducted using a monochromatic Al Kα source (hv = 1,486.6 eV) operating at 75 W with an X-ray beam size of 400 μm. Spectra were recorded at a pressure below 2.0 × 10^-7^ Torr, with calibration against the characteristic binding energy of C 1s at 284.6 eV.

**Proton nuclear magnetic resonance spectroscopy (^1^H NMR).** Proton nuclear magnetic resonance spectroscopy (^1^H NMR; AVANCE NEO 600; Bruker) spectra were acquired using a 5 mm Prodigy probe. ^1^H NMR spectra were recorded at an operating frequency of 600.25 MHz with a pulse length of 4 μs, a relaxation delay of 5 s, and 16 scans using the zg30 pulse sequence.

### Statistical Analysis

All experiments were performed in triplicate, and the data were averaged. Standard deviations were calculated in order to assess the variability in inhibition zone diameters observed in the disc diffusion assays. Group differences were evaluated by one-way ANOVA using the IBM SPSS Statistics software (ver. 29; SPSS Inc., USA). Significant differences between means were determined using Tukey’s test, with *p* < 0.05 considered significant.

## Results

### Assessment of Antibacterial Activity Using Disc Diffusion

Antibacterial activity was observed against *E. coli*, *L. monocytogenes*, *S. aureus*, and *S. typhimurium*. A buffer control with a pH of 2.3 was used to determine whether the antibacterial effects were due to the low pH of kombucha. No inhibition zones were observed with the buffer control, indicating that the antibacterial activity was intrinsic to the kombucha broth. Dried SCOBY exhibited a higher antibacterial effect than kombucha broth. Kombucha broth displayed the largest inhibition zone against *S. aureus*, with a diameter of 2.21 ± 0.01 cm, while the smallest zone was observed against *E. coli* at 1.44 ± 0.08 cm. For dried SCOBY, there was minimal variation among bacterial strains. However, *S. typhimurium* exhibited the largest inhibition zone, with a diameter of 2.22 ± 0.56 cm, while *S. aureus* showed the smallest, with a diameter of 2.03 ± 0.16 cm. *S. aureus* showed the largest inhibition zone in the kombucha broth, but the smallest in the dried SCOBY.

**Fig. 1:**
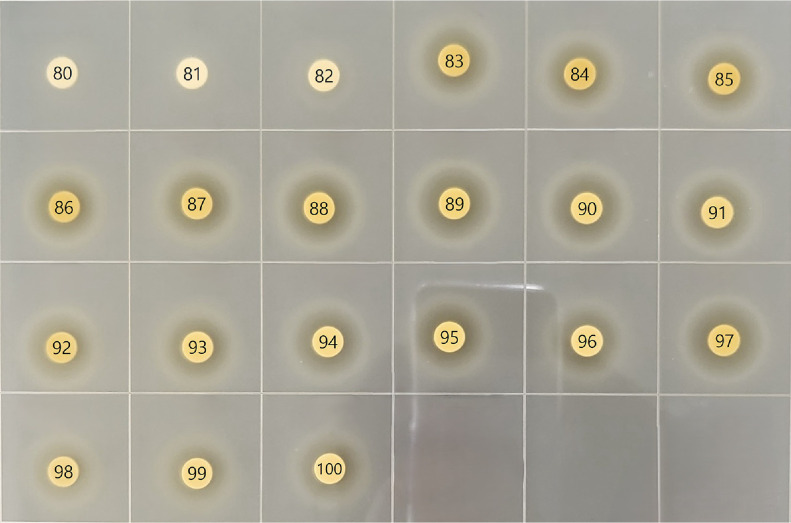
Antibacterial activity by disk diffusion analysis using silica gel chromatography separation of SCOBY extract.

When antibacterial compounds in the kombucha broth were separated using organic solvents via a separatory funnel, antibacterial activity was not confirmed (data not shown). Conversely, antibacterial activity was detected when compounds from the dried SCOBY were extracted with organic solvents, yielding inhibition zones of 1.08 ± 0.04 cm for *L. monocytogenes* and 1.03 ± 0.04 cm for *S. aureus* (Table 1). The SCOBY extract was further fractionated using silica gel column chromatography. Each fraction was then spotted (2 μl) onto a thin-layer chromatography (TLC) plate (Merck), and fractions displaying prominent spots under UV light at 254 nm were selected for antibacterial testing. Inhibition zones were observed for fractions 83–100. These active fractions were pooled and filtered, and the solvent was removed (Fig. 1).

### Instrumental Analysis of Antibacterial Compounds

**Fig. 2:**
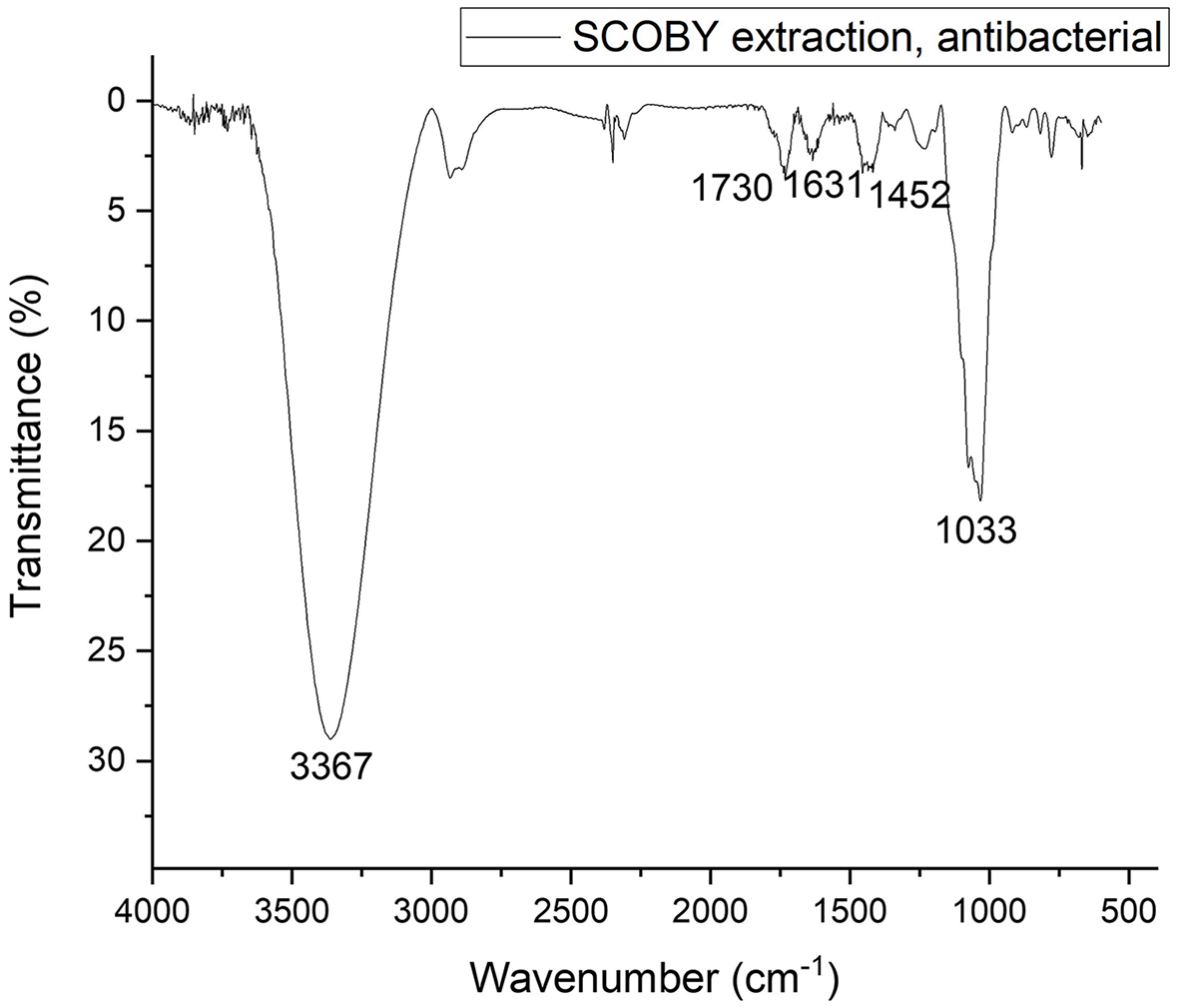
Fourier-transform infrared spectroscopy (FT-IR) spectra for the SCOBY extract fractions.

An OH absorption peak at 3,417 cm^-1^ was observed in the spectra of sample [18]. C-H stretching vibrations appeared at 2,937 cm^-1^ [19], while a sharp band at 1,632 cm^-1^ can be attributed to the C=O (carbonyl group) stretching vibration [20]. The peak at 1,059 cm^-1^ is due to C–O vibrations in the furan ring (Fig. 2) [21].

**Fig. 3:**
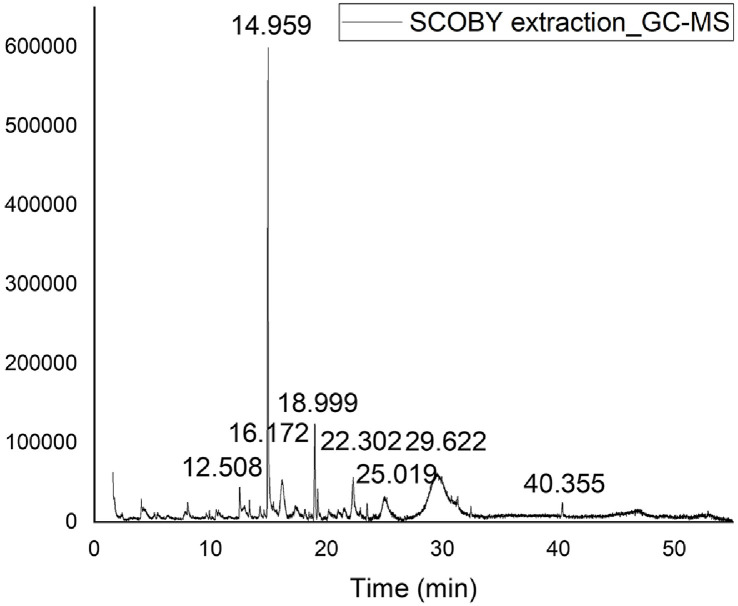
GC-MS spectra of antibacterial substitutes from SCOBY extract fractions.

Antibacterial compounds were identified based on retention indices and the mass spectra of these compounds were interpreted based on the National Institute of Standards and Technology (NIST) database. The GC analysis followed by comparison with the NIST database, identified nine compounds in the antibacterial substitute for the SCOBY extract. The chromatograms for these compounds are shown in Figure 3, and their detailed chemical compositions, retention times, molecular formulas, molecular weights, and concentrations are presented in Table 2 [22]. Among the identified compounds, HMF (molar mass = 126.11) exhibited the highest spectral similarity (93%) in comparison with the NIST database.

**Fig. 4:**
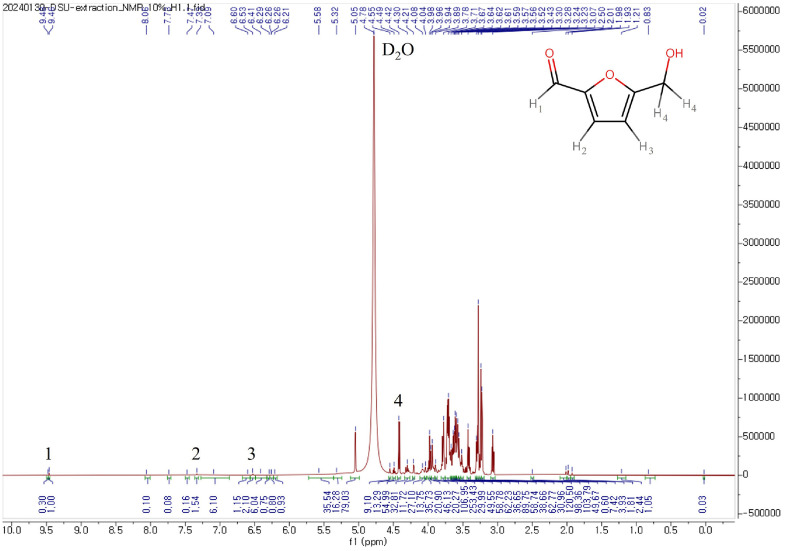
_1_H NMR spectra of antibacterial substitutes from SCOBY extract fractions.

A singlet from proton H1 was detected at 9.46 mg/l, with two doublets from the furanic ring protons (H2 and H3) observed at 7.33 and 6.53 mg/l, respectively. An additional signal at 4.49 mg/l, integrating for two protons, is due to H4. However, these signals are less suitable for identification of 4.49 mg/l due to overlap with those of other compounds (Fig. 4) [23].

**Fig. 5:**
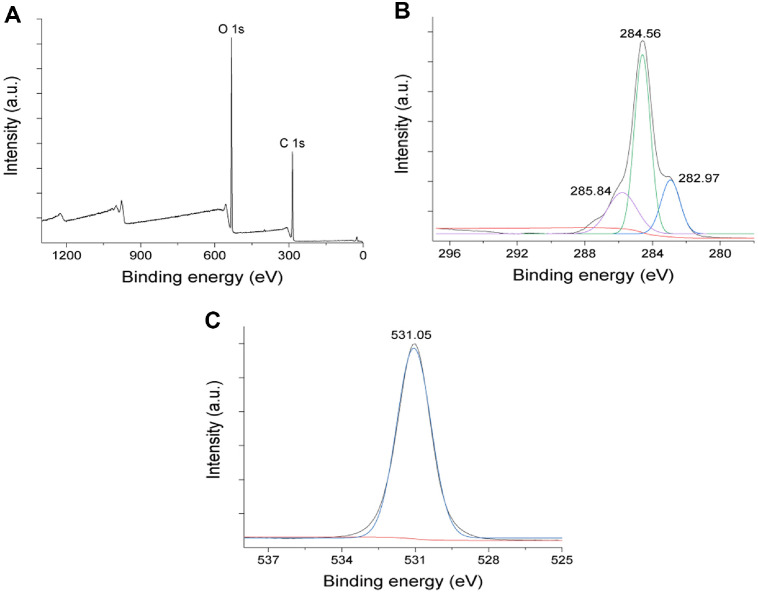
XPS spectra of antibacterial substitutes from SCOBY extract fractions. (**A**) Survey, (**B**) C1s, (**C**) O1s.

The XPS spectra revealed C1s at 285.87 eV and O1s at 532.32 eV as primary features, with binding energy calibration based on the C1s peak at 284.6 eV. Deconvolution of C1s showed surface functional groups at 282.97, 284.56, and 285.84 eV, which are assigned to the sp² bond (C=C), sp³ bond (C-C), and carbon–hydroxyl bond (C-OH), respectively, as shown in Figure 5B [24, 25]. Deconvolution of O1s showed functional groups at 531.04 and 532.78 eV, attributed to carbonyl oxygen (C=O) and hydroxyl oxygen (-OH), respectively, as shown in Figure 5C [26, 27].

The SCOBY exuretract fractions that demonstrated Antibacterial activity formed inhibition zones against the pathogenic bacteria *E. coli*, *L. monocytogenes*, *S. aureus*, and *S. typhimurium*. Chloramphenicol, which was used as a positive control, exhibited broader inhibition zones than the SCOBY extract fractions. Instrumental analysis of the extracts revealed a compound resembling HMF, whose concentration correlated with the size of the inhibition zones. *L. monocytogenes* and *S. aureus* showed greater inhibition with HMF than with chloramphenicol. No inhibition zones appeared with the blank disc (Table 3).

## Discussion

This study confirmed the antibacterial activity of both kombucha and SCOBY, as well as antibacterial substances isolated from SCOBY extract, whose structure and physicochemical properties were investigated using a combination of FT-IR, GC/MS, XPS, and ^1^H NMR analyses. FT-IR spectroscopy was used to identify key functional groups in the SCOBY extract, such as hydroxyl and carbonyl groups, indicating the presence of bioactive molecules [28] GC/MS analysis, which is widely utilized to profile antimicrobial metabolites in fermented matrices [29], was employed to separate and identify volatile and semi-volatile compounds. XPS analysis was performed to examine the surface chemical composition of the antibacterial compounds, which is a critical factor influencing antibacterial performance [30]. Finally, ^1^H NMR spectroscopy, which is used to characterize the molecular features of small organic molecules derived from microbial or thermal conversion processes [31], was conducted to confirm the structural identity of the major bioactive compound in the SCOBY extract.

The antibacterial substance derived from SCOBY was identified as HMF, which has a molecular weight of 126.11 g/mol. HMF is used in the production of polymers, solvents, and pharmaceuticals, and as a fuel additive [32], and is considered promising for mitigating global warming, as it is derived from renewable biomass and can replace fossil-based chemicals, contributing to reduced greenhouse gas emissions [33]. Research is ongoing into the efficient synthesis and bioconversion of HMF which can be used to produce bio-based plastics and fuel additives such as FDCA and DMF [34]. HMF is commonly produced through the Maillard reaction, a non-enzymatic browning process that occurs during food heating and fermentation, and during prolonged honey storage. Consequently, HMF can be found in honey, bread, dairy products, cereals, fruit juices, and heat-processed sugar-containing foods [35]. The sterilization and fermentation involved in kombucha production likely lead to the generation of HMF from fructose and glucose. While studies have indicated negative health effects of HMF, including mucosal and skin toxicity, cytotoxicity, chromosomal aberrations, and carcinogenicity, recent research suggests potential positive effects, such as antioxidant, anti-inflammatory, and anti-allergic properties [36, 37]. Although the detailed toxic mechanisms of HMF remain unclear, it is known to inhibit yeast growth and slow fermentation, likely by interfering with pyruvate metabolism and indirectly affecting pyruvate dehydrogenase activity [38]. Moreover, at concentrations of 16 mg/ml, HMF has been shown to exhibit antibacterial activity against *B. cereus*, *E. coli*, and *P. mirabilis* [39]. These findings support the observations in the present study that HMF, identified in kombucha, SCOBY, and SCOBY extract, contributes to the antibacterial activity observed against *E. coli*, *L. monocytogenes*, *S. aureus*, and *S. typhimurium*

## Conclusion

This study highlights the antibacterial potential of SCOBY and identifies HMF as its key bioactive compound. Despite previous concerns regarding its toxicity, HMF exhibited notable antibacterial activity. These findings support the potential of HMF as a promising alternative to conventional antibacterial compound and suggest that SCOBY-derived HMF can be used as a sustainable and effective antimicrobial agent.

## Figures and Tables

**Table 1:** Antibacterial activity of kombucha broth, dried SCOBY and SCOBY extracts. Unit (cm)

	^1^Inhibition zone diameter (cm)			
	^2^pH 2.3	^3^Kombucha	^4^Dried SCOBY	^5^SCOBY extract (33×)
*E. coli*	-	1.44 ± 0.08^a^	2.06 ± 0.29^a^	0.82 ± 0.01^a^
*L. monocytogenes*	-	1.75 ± 0.09^ab^	2.07 ± 0.3^a^	1.08 ± 0.04^b^
*S. aureus*	-	2.21 ± 0.01^c^	2.03 ± 0.16^a^	1.03 ± 0.04^b^
*S. typhimurium*	-	1.92 ± 0.26^bc^	2.22 ± 0.56^a^	0.81 ± 0.01^a^

^1^Inhibition zone diameter (mean values and standard deviations include wells of 8 mm diameter, and 10 mm for dried SCOBY). Each experiment was carried out in triplicate.

^2^To prepare the same pH as the kombucha broth, 8.5 g/l of acetic acid was sterilized and filtered.

^3^The kombucha sample used kombucha broth fermented for 21 days.

^4^The dried SCOBY sample was made from a SCOBY from kombucha fermented for 21 days, dried overnight at 95 degrees, and then punched to a diameter of 10 mm.

^5^The SCOBY extract sample was made by extracting the dried SCOBY with methanol and then removing the solvent to 33X.

**Table 2:** Chemical profiling and GC-MS analysis of SCOBY extract fractions.

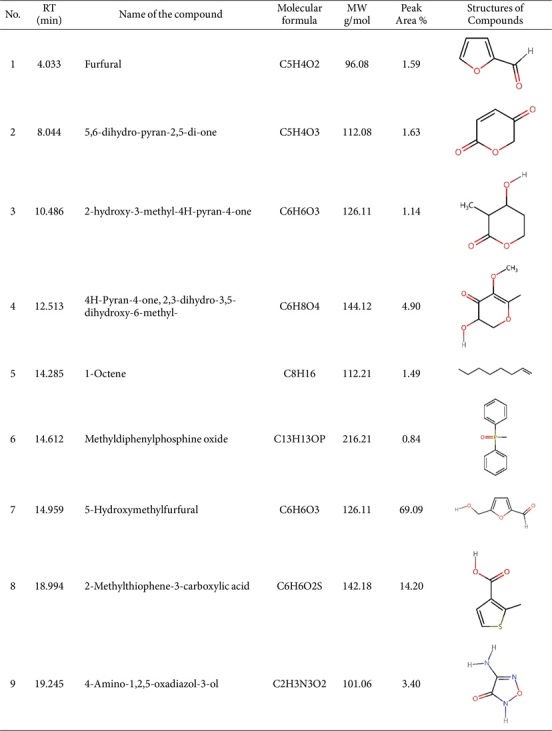

**Table 3:** The antibacterial activity against pathogenic bacteria. Unit (cm)

	^1^Inhibition zone diameter (cm)			
	^2^Blank	^3^SF	^4^HMF	^5^C
*E. coli*	-	1.48 ± 0.21^a^	2.36 ± 0.4^b^	1.8 ± 0.36^a^
*L. monocytogenes*	-	1.47 ± 0.06^a^	1.65 ± 0.17^a^	1.93 ± 0.06^a^
*S. aureus*	-	1.47 ± 0.06^a^	2.07 ± 0.15^ab^	1.45 ± 0.05^a^
*S. Typhimarium*	-	1.55 ± 0.06^a^	2.08 ± 0.16^ab^	2.11 ± 0.53^a^

^1^Inhibition zone diameter (mean values and standard deviations include wells of 8 mm diameter). Each experiment was carried out in triplicate.

^2^The blank sample used only a paper disc.

^3^The SF sample is SCOBY extract fractions of 35 μl/disc.

^4^The HMF sample is hydroxymethylfurfural 20 mg/disc.

^5^The C sample is chloramphenicol 30μg/disc.

## References

[1] Beović B (2006). The issue of antimicrobial resistance in human medicine. Int. J. Food Microbiol..

[2] Im DY, Lee KI (2011). Antioxidative and antibacterial activity and tyrosinase inhibitory activity of the extract and fractions from *Taraxacum coreanum* Nakai. Korean J. Med. Crop Sci..

[3] Maliyekkal SM, Lisha KP, Pradeep T (2010). A novel cellulose-manganese oxide hybrid material by in situ soft chemical synthesis and its application for the removal of Pb (II) from water. J. Hazard. Mater..

[4] Martínez Leal J, Valenzuela Suárez L, Jayabalan R, Huerta Oros J, Escalante-Aburto A (2018). A review on health benefits of kombucha nutritional compounds and metabolites. CyTA-J. Food.

[5] Anantachoke N, Duangrat R, Sutthiphatkul T, Ochaikul D, Mangmool S (2023). Kombucha beverages produced from fruits, vegetables, and plants: a review on their pharmacological activities and health benefits. Foods.

[6] Sreeramulu G, Zhu Y, Knol W (2000). Kombucha fermentation and its antimicrobial activity. J. Agric. Food Chem..

[7] Yan JH, Zheng DW, Gu HY, Yu YJ, Zeng JY, Chen QW (2023). In Situ Sprayed Biotherapeutic Gel Containing Stable Microbial Communities for Efficient Anti‐Infection Treatment. Adv. Sci..

[8] Kaewkod T, Bovonsombut S, Tragoolpua Y (2019). Efficacy of kombucha obtained from green, oolong, and black teas on inhibition of pathogenic bacteria, antioxidation, and toxicity on colorectal cancer cell line. Microorganisms.

[9] Hou J, Luo R, Ni H, Li K, Mgomi FC, Fan L (2021). Antimicrobial potential of kombucha against foodborne pathogens: a review. Qual. Assur. Saf. Crops Foods.

[10] Battikh H, Bakhrouf A, Ammar E (2012). Antimicrobial effect of Kombucha analogues. LWT-Food Sci. Technol..

[11] Salama A, Abouzeid RE, Owda ME, Cruz-Maya I, Guarino V (2021). Cellulose-silver composites materials: preparation and applications. Biomolecules.

[12] Bryszewska MA, Pareja DG, Kaczmarek L, Sobczyk-Guzenda A, Piotrowska M, Batory D (2024). SCOBY cellulose-based materials hydrophobized using stearic acid and apple powder. Int. J. Mol. Sci..

[13] Gorgieva S, Trček J (2019). Bacterial cellulose: production, modification and perspectives in biomedical applications. Nanomaterials.

[14] Oliveira TJ, Segato TCM, Machado GP, Grotto D, Jozala AF (2022). Evolution of bacterial cellulose in cosmetic applications: an updated systematic review. Molecules.

[15] Kim H, Shin M, Ryu S, Yun B, Oh S, Park D-J (2021). Evaluation of probiotic characteristics of newly isolated lactic acid bacteria from dry-aged Hanwoo beef. Food Sci. Anim. Resour..

[16] Hudzicki J (2009). Kirby-Bauer disk diffusion susceptibility test protocol. Am. Soc. Microbiol..

[17] Al Rashdi RSY, Hossain MA, Al Touby SSJ (2021). Antioxidant and antibacterial activities of leaves crude extracts of *Adenium obesum* grown in Oman National Botanical Garden. Adv. Biomarker Sci. Technol..

[18] Kanta U-a, Thongpool V, Sangkhun W, Wongyao N, Wootthikanokkhan J (2017). Preparations, characterizations, and a comparative study on photovoltaic performance of two different types of graphene/TiO2 nanocomposites photoelectrodes. J. Nanomater..

[19] Ogunkanmi J, Kulla D, Omisanya N, Sumaila M, Obada D, Dodoo-Arhin D (2018). Extraction of bio-oil during pyrolysis of locally sourced palm kernel shells: effect of process parameters. Case Stud. Therm. Eng..

[20] Surendra B, Veerabhadraswamy M (2017). Microwave assisted synthesis of polymer via bioplatform chemical intermediate derived from Jatropha deoiled seed cake. J. Sci. Adv. Mater. Devices.

[21] Belmokhtar A, Yahiaoui A, Hachemaoui A, Abdelghani B, Sahli N, Belbachir M (2012). A novel poly {(2, 5‐diyl furan)(benzylidene)}: a new synthetic approach and electronic properties. Int. Sch. Res. Notices.

[22] Chikowe I, Bwaila KD, Ugbaja SC, Abouzied AS (2024). GC-MS analysis, molecular docking, and pharmacokinetic studies of Multidentia crassa extracts' compounds for analgesic and anti-inflammatory activities in dentistry. Sci. Rep..

[23] Serra-Cayuela A, Castellari M, Bosch-Fusté J, Riu-Aumatell M, Buxaderas S, López-Tamames E (2013). Identification of 5-hydroxymethyl-2-furfural (5-HMF) in Cava sparkling wines by LC-DAD-MS/MS and NMR spectrometry. Food Chem..

[24] Shi Q, Hou C, Wang H, Zhang Q, Li Y (2015). Rapid formation of superelastic 3D reduced graphene oxide networks with simultaneous removal of HI utilizing NIR irradiation. J. Mater. Chem. A..

[25] Kuwabara A, Kuroda S-i, Kubota H (2007). Polymer surface treatment by atmospheric pressure low temperature surface discharge plasma: its characteristics and comparison with low pressure oxygen plasma treatment. Plasma Sci. Technol..

[26] Duguet T, Bessaguet C, Aufray M, Esvan J, Charvillat C, Vahlas C (2015). Toward a computational and experimental model of a poly-epoxy surface. Appl. Surface Sci..

[27] Stypczyńska A, Nixon T, Mason N (2014). X-ray radiation of poly-L-arginine hydrochloride and multilayered DNA-coatings. Eur. Phys. J. D..

[28] Ferdous J, Qais FA, Ali F, Palit D, Hasan I, Kawsar SM (2024). FTIR, 1H-/13C-NMR spectral characterization, antimicrobial, anticancer, antioxidant, anti-inflammatory, PASS, SAR, and in silico properties of methyl α-D-glucopyranoside derivatives. Chem. Phys. Impact.

[29] Soni K, Bagaria A (2024). GC-MS based identification of anti-microbial bioactive compounds, isolated from *Bacillus halotolerans* of marine sediment. J. Exper. Mar. Biol. Ecol..

[30] Al-Gaashani R, Zakaria Y, Gladich I, Kochkodan V, Lawler J (2022). XPS, structural and antimicrobial studies of novel functionalized halloysite nanotubes. Sci. Rep..

[31] Saadouli I, Zendah El Euch I, Trabelsi E, Mosbah A, Redissi A, Ferjani R (2020). Isolation, characterization and chemical synthesis of large spectrum antimicrobial cyclic dipeptide (L-leu-L-pro) from *Streptomyces misionensis* V16R3Y1 bacteria extracts. A novel 1H NMR metabolomic approach. Antibiotics.

[32] Meier S (2020). Mechanism and malleability of glucose dehydration to HMF: entry points and water-induced diversions. Catal. Sci. Technol..

[33] Nomura T, Minami E, Kawamoto H (2021). Hydroxymethylfurfural as an intermediate of cellulose carbonization. ChemistryOpen.

[34] Cong H, Yuan H, Tao Z, Bao H, Zhang Z, Jiang Y (2021). Recent advances in catalytic conversion of biomass to 2, 5-furandicarboxylic acid. Catalysts.

[35] Bastos DM, Monaro É, Siguemoto É, Séfora M. 2012. *Maillard reaction products in processed food: pros and cons*, pp. 281-300. Ed. INTECH Open Access Publisher London.

[36] Shapla UM, Solayman M, Alam N, Khalil MI, Gan SH (2018). 5-Hydroxymethylfurfural (HMF) levels in honey and other food products: effects on bees and human health. Chem. Cent. J..

[37] Zhang H, Jiang Z, Shen C, Zou H, Zhang Z, Wang K (2021). 5-Hydroxymethylfurfural alleviates inflammatory lung injury by inhibiting endoplasmic reticulum stress and NLRP3 inflammasome activation. Front. Cell Dev. Biol..

[38] Almeida JR, Röder A, Modig T, Laadan B, Lidén G, Gorwa-Grauslund M-F (2008). NADH-vs NADPH-coupled reduction of 5-hydroxymethyl furfural (HMF) and its implications on product distribution in *Saccharomyces cerevisiae*. Appl. Microbiol. Biotechnol..

[39] Duru CE, Duru IA, Nwagbara NK (2012). Potency of 5-hydroxymethylfurfuraldehyde (HMF) against *Bacillus cereus* and *Proteus mirabilis*. Biochem. Indian J..

